# How ‘core’ are motor timing difficulties in ADHD? A latent class comparison of pure and comorbid ADHD classes

**DOI:** 10.1007/s00787-015-0734-0

**Published:** 2015-07-08

**Authors:** Jolanda M. J. van der Meer, Catharina A. Hartman, Andrieke J. A. M. Thissen, Anoek M. Oerlemans, Marjolein Luman, Jan K. Buitelaar, Nanda N. J. Rommelse

**Affiliations:** Department of Cognitive Neuroscience, Donders Institute for Brain, Cognition and Behavior, Radboud University Nijmegen Medical Center, Reinier Postlaan 12, 6525 GC, Nijmegen, The Netherlands; Karakter Child and Adolescent Psychiatry University Center Nijmegen, Nijmegen, The Netherlands; Department of Psychiatry, University of Groningen Medical Centre, Groningen, The Netherlands; Department of Psychiatry, Donders Institute for Brain, Cognition and Behavior, Radboud University Nijmegen Medical Center, Nijmegen, The Netherlands; Department of Clinical Neuropsychology, VU University Amsterdam, Amsterdam, The Netherlands

**Keywords:** ADHD, ASD, Latent class analyses (LCA), Motor timing, Variability

## Abstract

Children with attention-deficit/hyperactivity disorder (ADHD) have motor timing difficulties. This study examined whether affected motor timing accuracy and variability are specific for ADHD, or that comorbidity with autism spectrum disorders (ASD) contributes to these motor timing difficulties. An 80-trial motor timing task measuring accuracy (*μ*), variability (*σ*) and infrequent long response times (*τ*) in estimating a 1-s interval was administered to 283 children and adolescents (8–17 years) from both a clinic and population based sample. They were divided into four latent classes based on the SCQ and CPRS-R:L data. These classes were: without behavioral problems ‘Normal-class’ (*n* = 154), with only ADHD symptoms ‘ADHD-class’ (*n* = 49), and two classes with both ASD and ADHD symptoms; ADHD(+ASD)-class (*n* = 39) and ASD(+ADHD)-class (*n* = 41). The pure ADHD-class did not deviate from the Normal class on any of the motor timing measures (mean RTs 916 and 925 ms, respectively). The comorbid ADHD(+ASD) and ASD(+ADHD) classes were significantly less accurate (more time underestimations) compared to the Normal class (mean RTs 847 and 870 ms, respectively). Variability in motor timing was reduced in the younger children in the ADHD(+ASD) class, which may reflect a tendency to rush the tedious task. Only patients with more severe behavioral symptoms show motor timing deficiencies. This cannot merely be explained by high ADHD severity with ASD playing no role, as ADHD symptom severity in the pure ADHD-class and the ASD(+ADHD) class was highly similar, with the former class showing no motor timing deficits.

## Introduction

Attention deficit/hyperactivity disorder (ADHD) is a neurodevelopmental disorder that is typified by developmentally inappropriate degrees of inattention, impulsivity, and hyperactivity [1]. Broad patterns of neuropsychological impairments have been associated with ADHD, among which are the deficits in time processing [2, 3]. Falter and Noreika suggested that deficits in time processing may play an important role in neurodevelopmental disorders like ADHD by interacting with primary symptoms [4]. For example, previous studies suggest that difficulties in complex functions such as attention, language, and inhibition are associated with reduced time processing, as these complex functions are characterized by specific temporal patterns [5, 6]. Time processing can be measured with a motor timing paradigm in which the accuracy and variability of motor timing, and infrequent long response times are disentangled by using *μ*, *σ*, and *τ*. These measures may differentially affect cognitive functions that rely on accurate motor timing [7–9]. Reduced motor time processing has frequently been associated with ADHD, despite systematic differences across studies, and has shown to be highly heritable, suggestive of an etiological role in ADHD [2, 10–13]. Of note, abnormalities in motor timing are predominantly related to deficient motor *timing* processes rather than to general deficient *motor* functioning in children and adolescents who suffer from ADHD [14].

Despite this compelling evidence for motor timing difficulties in ADHD, reduced time processing has not exclusively been found in ADHD. It has also been observed in other disorders including autism spectrum disorders (ASD) [15–18]. ADHD is frequently comorbid with ASD; in clinical samples, 20–50 % of ADHD patients meet criteria for ASD (for review see [19]). Therefore, it remains to be seen whether difficulties in motor time processing can be found in ‘pure’ ADHD, or rather are associated with comorbid ASD [19–24]. For example, Adamo and colleagues compared response time variability in normally developing children, children with ADHD, and children with ASD with and without substantial comorbid ADHD symptoms [24]. Their findings suggest that both children with ADHD and children with ASD and comorbid ADHD had elevated levels of response time variability. In contrast, children with ASD without substantial comorbid ADHD symptoms did not differ from normally developing children regarding response time variability, suggesting that response time variability is more strongly related to ADHD. However, in addition to subtyping along traditional lines of DSM-based categories, a comparison of (motor) timing across homogeneous subgroups within comorbid ASD-ADHD children may be a powerful method to further our understanding of both disorders. Such homogeneous subgroups based on quantitative symptom measures reflect the continuously distributed nature and severity of ASD and ADHD symptoms across the general population, as shown by several studies [25–29].

We previously reported on the advantages of more homogeneous subgroups of comorbid ASD–ADHD children when studying shared substrates in a clinic and population-based sample [30]. In that study, classes were derived using latent class analyses (LCA), an empirical method which allows classifications based on the type and severity of ASD and ADHD symptoms. We showed that ADHD symptoms were present both in the absence and presence of ASD symptoms. This resulted in a pure ADHD class that showed no comorbid symptoms of ASD, and an ADHD class with comorbid ASD (ADHD(+ASD)). Furthermore, ASD symptoms were reported in the presence of less severe ADHD symptoms (ASD(+ADHD)), but no class with pure ASD behavior was identified. The empirical validity of these distinct classes was affirmed by the overlap and distinctiveness of associated comorbidity patterns and cognitive profiles. Classes with children suffering from both types of symptoms were overall cognitively more impaired than children with only ADHD symptoms, indicative for an overlapping cognitive background in ASD and ADHD. Importantly, cognitive specificity was found in that ADHD(+ASD) class which showed the more typical ADHD neurocognitive problems (working memory deficits), while the ASD(+ADHD) class showed more typical ASD neurocognitive problems (emotion recognition problems and superior block pattern performance). This cognitive double dissociation between comorbid classes with either more profound ASD or more profound ADHD symptoms can increase our understanding of the distinct substrates for ASD and ADHD.

The cognitive domain of time processing is an additional candidate for furthering our understanding of these more homogeneous subgroups of children affected with pure ADHD or affected with both ASD and ADHD symptomatologies. The current study was set out to examine the overlap and distinctiveness in motor timing abilities between these homogeneous subgroups with the use of a well-validated motor timing paradigm [9, 14, 31]. This paradigm measures the accuracy, variability, and infrequent long response times of 1 s interval motor time productions with the use of the parameters *μ*, *σ*, and τ, respectively. In sum, the aims were to examine whether the (1) accuracy, (2) variability, and (3) infrequent long response times differed across the four homogeneous ADHD-ASD symptom classes. Given the previous findings in more homogeneous subgroups [30], we hypothesized that motor timing is affected (i.e., reduced accuracy, increased variability of motor timing, and increased infrequent long response times) in classes with both ADHD and ASD symptoms, and to a lesser extent, although still different from the Normal class, in the pure ADHD class.

## Methods

### Participants

The task was randomly assigned to 283 participants between 8 and 17 years of age from a population- and clinic-based sample. This sample originally consisted of 644 participants [30]; because of task demands, the current task was not administered to the 5, 6, and 7 year olds. Eighty-one children originated from a random population cohort study [Schoolkids Project Interrelating DNA and Endophenotype Research (SPIDER)] and 202 children and adolescents from a clinical ASD–ADHD genetic study [Biological Origins of Autism (BOA)]. The BOA cohort consisted of patients with DSM-IV based ASD, ADHD, and ASD+ADHD diagnoses and non-affected siblings (for a full description, see [32]).

In the previous study, participants were divided in homogeneous symptom classes with the use of a LCA on the raw subscale outcomes of the SCQ (Social Interaction, Communication and Stereotypic Behavior) and the *T*-scores of the following ten scales of the CPRS-R:L: social problems, inattention, restlessness, cognitive problems, hyperactivity, oppositional behavior, emotional lability, anxiety, perfectionism, and psychosomatic complaints (for a full description, see [30]). The raw subscale outcomes of the SCQ and the *T*-scores of the CPRS used were either unrelated to age (SCQ) or corrected for the influence of age (CPRS), limiting the impact of age on the definition of the latent classes. Five classes had the best fitting BIC and SSA BIC values and entropy (0.914), combined with informative class profiles [33]. Between class contrasts indicated that the current subsample was comparable to the complete sample regarding ASD symptom severity (all *p*’s > 0.06), ADHD symptom severity (all *p*’s > 0.08), sex (all *p*’s > 0.21), and IQ (all *p*’s > 0.21). Consequently, the current sample was older (*M* (SD) 11.57 (2.5)) than the complete sample (*M* (SD) 9.5 (2.4)). The distribution of children across the distinct homogeneous symptom classes, as well as the ASD and ADHD symptom severity, age, sex, population, and IQ distributions are provided in Table 1. These distributions are well in line with the distributions in the complete sample [30].

**Table 1:** Demographic characteristics of the children in the distinct classes

	Normal		ADHD^a^		ADHD(+ASD)^a^		ASD(+ADHD)^a^		Contrasts based on *p*-values of 0.05
	*n* = 154		*n* = 49		*n* = 39		*n* = 41		
	*M*	SD	*M*	SD	*M*	SD	*M*	SD	
Age in years	11.2	2.3	11.6	2.5	12.3	2.7	12.1	2.5	Normal < ADHD < ADHD(+ASD) = ASD(+ADHD)
% Male	42.2		69.4		79.5		85.4		Normal < ADHD = ADHD(+ASD) = ASD(+ADHD)
% Population based^b^	39.0		30.6		15.4		0.0		
Estimated full-scale IQ^c^	106.7	12.1	104.7	11.8	100.5	12.5	102.2	10.6	Normal > ADHD(+ASD)
Total score SCQ^d^	4.3	5.4	8.2	5.2	16.4	6.6	22.4	6.0	Normal < ADHD < ADHD(+ASD) < ASD(+ADHD)
*T*-score CPRS Inattention^e^	48.2	6.5	65.0	7.1	73.3	8.8	62.5	8.6	Normal < ADHD = ASD(+ADHD) < ADHD(+ASD)
*T*-score CPRS hyperactive-impulsive^e^	48.4	7.2	66.3	10.6	79.9	8.8	67.1	12.4	Normal < ADHD = ASD(+ADHD) < ADHD(+ASD)
*T*-score CPRS oppositional behavior^e^	48.3	6.8	57.22	8.3	74.5	9.0	59.4	10.9	Normal < ADHD = ASD(+ADHD) < ADHD(+ASD)
*T*-score CPRS cognitive problems^e^	49.0	6.5	65.0	8.0	71.8	7.6	59.7	8.3	Normal < ASD (+ADHD) < ADHD < ADHD (+ASD)
*T*-score CPRS anxiety^e^	50.7	11.1	55.4	11.4	71.5	13.6	69.6	13.1	Normal = ADHD < ASD(+ADHD) = ADHD(+ASD)
*T*-score CPRS perfectionism^e^	46.6	6.4	49.7	6.4	60.5	11.3	64.8	9.9	Normal = ADHD < ADHD(+ASD) = ASD(+ADHD)
*T*-score CPRS psychosomatic complaints^e^	50.7	9.7	54.7	11.8	72.1	15.6	63.0	14.6	Normal = ADHD < ASD(+ADHD) < ADHD(+ASD)
*T*-score CPRS emotional lability^e^	46.1	7.1	54.5	10.7	71.3	13.9	58.3	10.5	Normal < ADHD = ASD(+ADHD) < ADHD(+ASD)

^a^ ADHD = class with behavioral problems in ADHD only. ADHD(+ASD) = class with severe ADHD symptoms, who also show ASD symptoms. ASD(+ADHD) = class with severe ASD symptoms, who also show ADHD symptoms

^b^ Percentage of the class derived from the general population

^c^ Full-scale IQ was estimated by four subtests of the WPPSI, WISC-III, or WAIS-III: Block Design, Picture Completion, Similarities, and either Vocabulary or Arithmetic [34, 35]. These subtests are known to correlate 0.90–0.95 with Full-scale IQ [36]

^d^ The total score on the Social Communication Questionnaire (SCQ) reflected the total amount of ASD symptoms. The official cut-off score for probable ASD is 15, and for definite ASD the cut-off is 21

^e^ *T*-scores on the CPRS (Conners’ Parent Rating Scale) subscales reflected the degree of ADHD-related and comorbid symptoms. The official cut-off for clinically relevant symptoms on the CPRS is a *T*-score above 63

For the sake of clarity, the classes were labeled. Children in class ‘Normal’ showed hardly any problems on ASD and ADHD behavioral domains (*n* = 154). Next, class ‘ADHD’ contained children with only ADHD symptoms (*n* = 49) without comorbidities. Here both DSM-IV-oriented CPRS subscales for ADHD (Inattentive and Hyperactive-Impulsive behavior) were above clinical cut-off, whereas the SCQ total score was substantially below cut-off (see Table 1). Children in the class ‘ADHD(+ASD)’ scored above clinical cut-off on both ADHD and ASD symptoms, with the ADHD symptoms more prominent than the ASD symptoms (*n* = 39). Finally, children in the class ‘ASD(+ADHD)’ scored at/above clinical cut-off on both ADHD and ASD symptoms, with the ASD symptoms more prominent than the ADHD symptoms (*n* = 41). No class with only ASD behavior was identified. All children were of Caucasian descent and had an estimated total IQ of at least 70 on the Wechsler Intelligence Scale (WISC-III) or Wechsler Adult Intelligence Scale (WAIS-III) [34, 35]. Exclusion criteria were epilepsy, known genetic or chromosomal disorders (such as Down syndrome), brain damage, and problems with vision or hearing.

### Measures

#### Motor timing task

This 1 s time production task measured the accuracy, variability, and infrequent long response times of (motor) timing [9, 14, 28]. Participants had to press a button with their preferred index finger when they thought a 1-s time interval had elapsed. The start of the interval was announced by a tone. After the button press, visual feedback concerning the accuracy of the response was presented on screen, indicating whether the response was correct, too fast or too slow. A response was regarded correct when it fell between the lower and upper boundary set by a dynamic (self-paced) tracking algorithm. Boundaries were set at 500–1500 ms at the beginning of the task [28]. If the response was within these boundaries, the boundaries of the subsequent trial was narrowed by 100 ms. Likewise, the boundaries of the subsequent trial were widened with 100 ms if the response on the previous trial fell outside the boundaries. The practice session consisted of 20 trials and the experimental session of 80 trials.

Accuracy of motor timing was represented by *μ* and the mean of time productions in ms, corrected for the tail of the distribution (infrequent long response times). Variability in timing was represented by *σ* and the variability of motor time productions in ms, corrected for the tail of the distribution (infrequent long response times). Infrequent long response times were represented by *τ*, the mean of the exponential part of the distribution [8]. Dependent measures *μ* (mu), *σ* (sigma), and *τ* (tau) were calculated with the use of ex-Gaussian analyses performed in MATLAB.

### Procedure

The task described was part of the broader neuropsychological assessment batteries used in the SPIDER and BOA projects. These studies have been approved by the Committee on Research involving Human Subjects (CMO) and participants were enrolled between January 2009 and July 2011. After complete description of the study to the parents and adolescents, written informed consent was obtained. Parents were invited to fill in several questionnaires concerning their youngster’s behavior.

### Data analyses

Raw responses higher or lower than 4 SD from a subject’s mean, with a minimum response time of 200 ms, were considered outliers and excluded, which was <0.1 % of the data [8]. Slow responses <4 SD below a subject’s mean were not excluded, but represented by *τ*, the mean of the exponential part of the distribution. Since *τ*-data were positively skewed, normalized *z*-scores for *τ* were used in all analyses. These *z*-scores were obtained by Van der Waerden transformations (SPSS version 20). Effect sizes were defined in terms of percentage of variance explained (*η*_*p*_^2^). Small, medium, and large effects were defined as explained variances of 0.01, 0.06, and 0.14, respectively [37].

The classes were compared using Repeated Measures ANCOVAs with class membership and sex as a fixed factor, age, and age^2^ as covariates. Dependent variables were *μ*, *σ*, and *τ*, respectively. Age^2^ was included to adjust for possible nonlinear improvement in tasks performance across age. Interaction effects were examined and, if nonsignificant, dropped from the model. Post hoc analyses were repeated with IQ and the measures for ADHD, oppositional behavior, emotional lability, anxiety, perfectionism, and psychosomatic complaints as covariates to examine whether results changed when correcting for between-classes differences in these domains. Correction for multiple comparisons was applied according to the false discovery rate (FDR) controlling procedure to the post hoc analyses with a *p* value setting of 0.05 [38]. Only the effects that remained significant after FDR-correction were reported. Finally, in light of possible cognitive impairments in unaffected siblings, analyses were repeated excluding unaffected siblings of ASD, ADHD, and ASD+ADHD-affected participants in the Normal class, to examine a potential influence on the findings.

## Results

### Accuracy (*μ*)

**Fig. 1:**
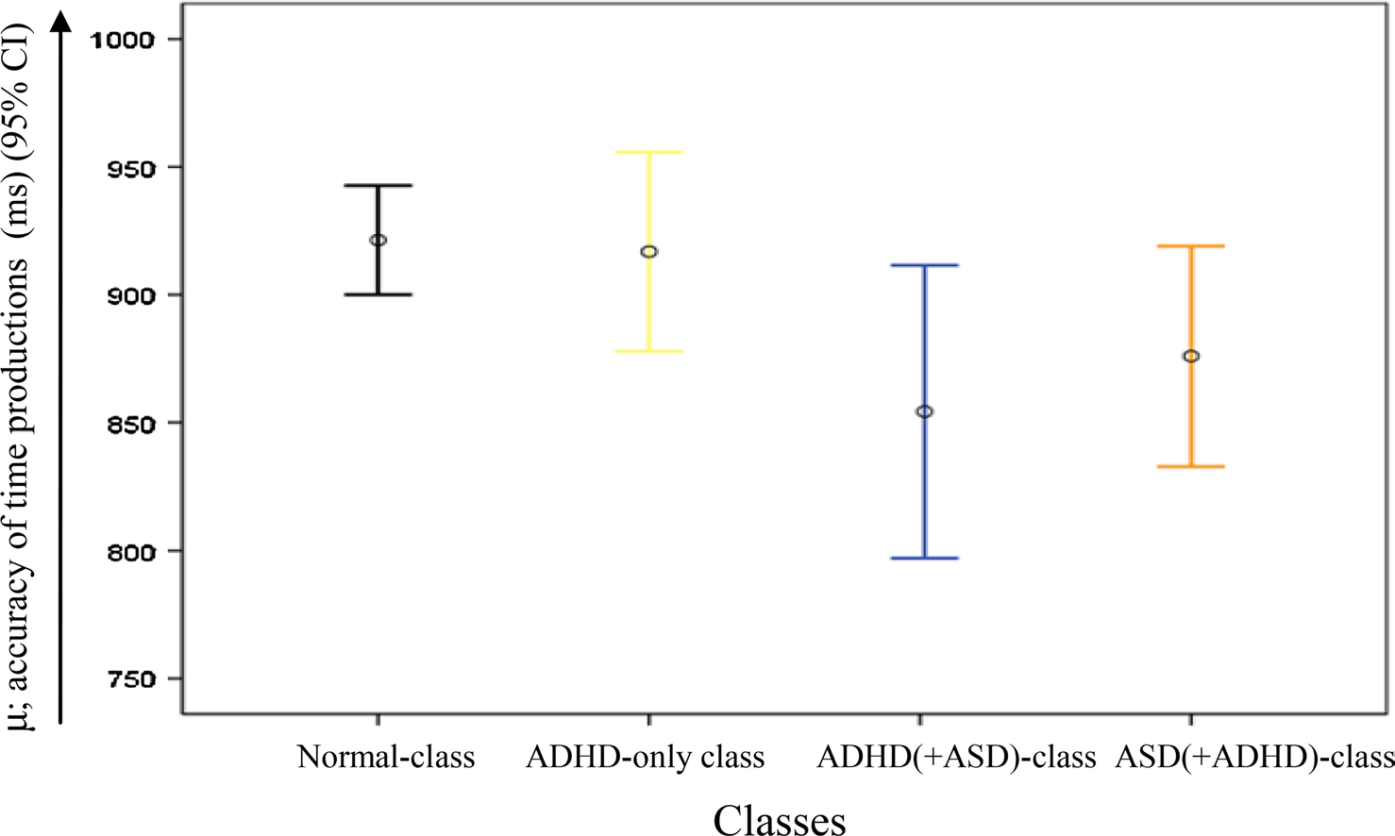
The accuracy of time productions (ms) corrected for infrequent long response times in the distinct classes

A significant *class* effect, however with small effect size, was found for *μ* (*F*(3282) = 4.20, *p* = 0.006, *η*_*p*_^2^ = 0.04). All classes seem to underproduce the 1-s interval (see Fig. 1). Pairwise comparisons indicated that the deviation from the aimed response time (1000 ms) of the ADHD(+ASD) (*M* = 847 ms) and ASD(+ADHD) (*M* = 870 ms) classes deviated significantly from that of the Normal class (*M* = 925 ms) (*p* = 0.002 and *p* = 0.025, respectively), while the accuracy of the only ADHD class did not differ from that of the other classes (*M* = 916 ms). No significant *class by age* interaction effect was found for *μ* (*F*(3282) = 1.71, *p* = 0.16, *η*_*p*_^2^ = 0.02). A significant positive linear *age* effect, however with small effect size, was found for *μ* (*F*(1282) = 10.37, *p* = 0.001, *η*_*p*_^2^ = 0.04), with more accurate responses in older than younger children.

### Variability (*σ*)

**Fig. 2:**
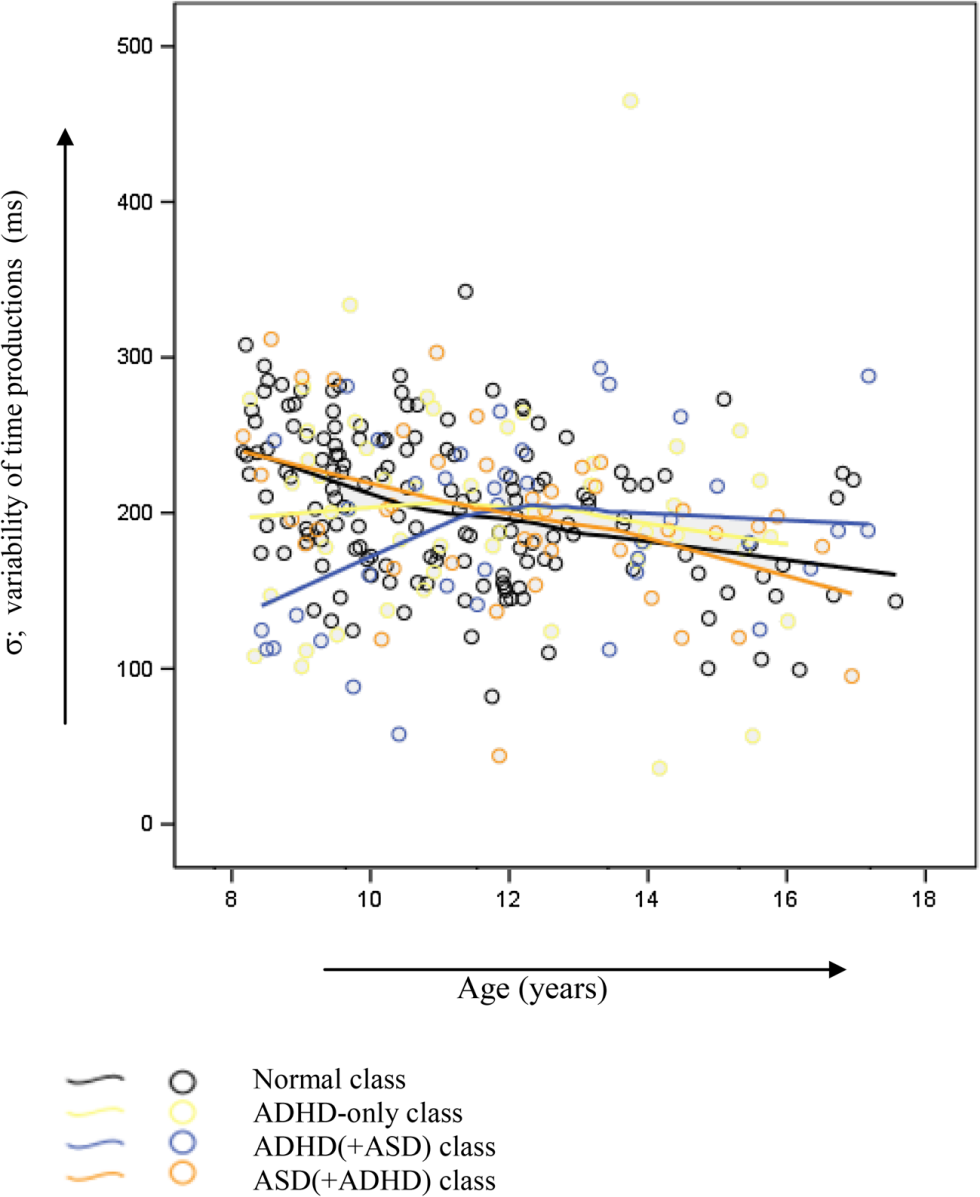
The variability of time productions (ms) corrected for infrequent long response times across age in the distinct classes

No significant *class* effect was found for the *σ* (*F*(3282) = 0.86, *p* = 0.46, *η*_*p*_^2^ = 0.01). A significant *class by age* interaction effect with a medium effect size, was found for *σ* (*F*(3282) = 5.58, *p* = 0.001, *η*_*p*_^2^ = 0.06), see also Fig. 2. Post hoc analysis including two age groups per class indicated that younger children in all classes except for the ADHD(+ASD) class showed more variability compared to their older counterparts. A mean split for *μ* in the ADHD(+ASD) class indeed indicated 9.8 % lower mean response times for less variable children (*M* = 809.72) compared to their more variable counterparts (*M* = 897.56).

### Infrequent long response times (*τ*)

**Fig. 3:**
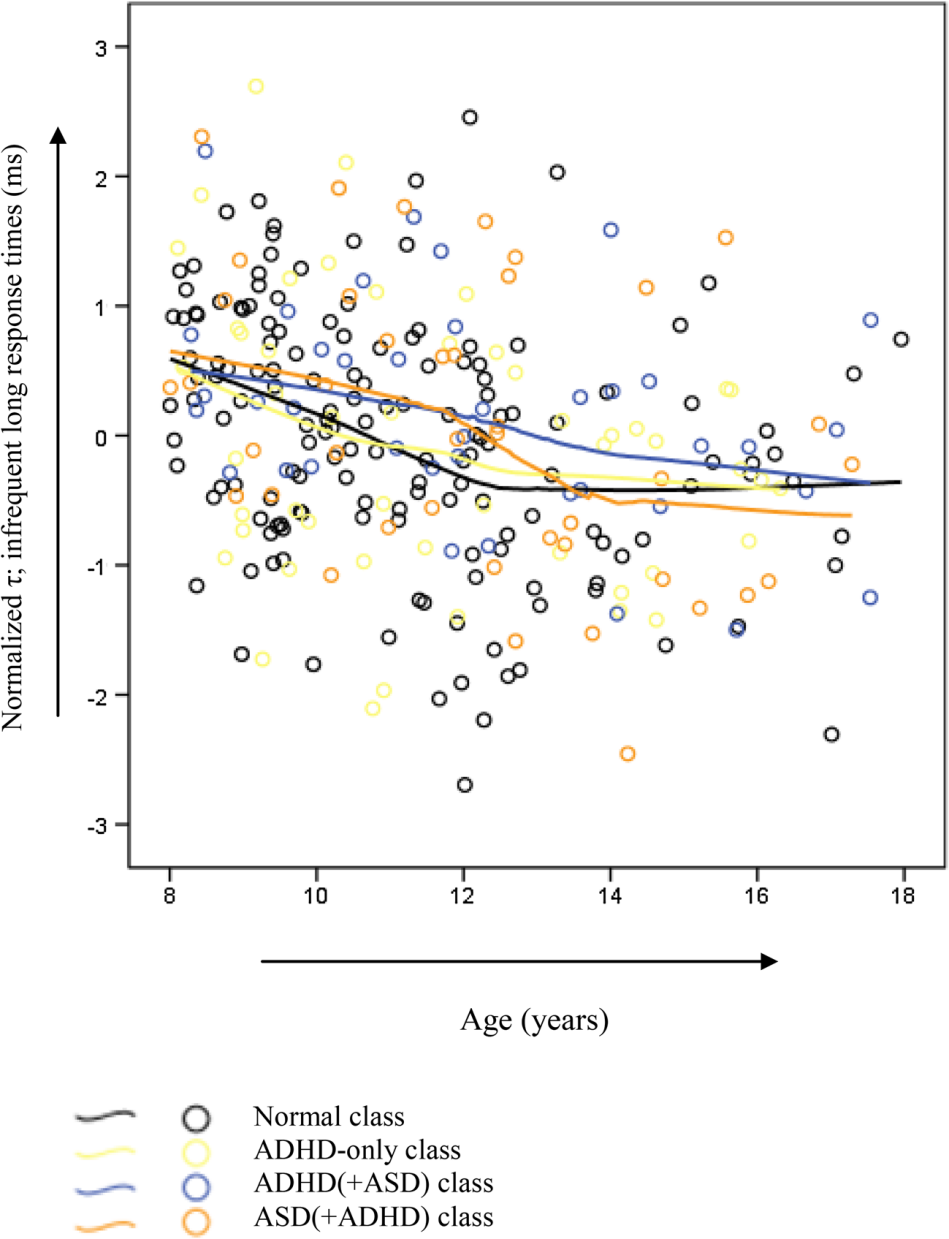
Infrequent long response times (ms) across age in the distinct classes

No significant *class* effect nor significant *class by age* interaction effect was found for the *τ* (*F*(3282) = 1.53, *p* = 0.21, *η*_*p*_^2^ = 0.02 and *F*(3282) = 0.20, *p* = 0.90, *η*_*p*_^2^ = .00, respectively). A significant positive linear effect of *age* with medium effect size and a significant effect of *age*^2^ with a small effect size were found for these infrequent long response times (*F*(1282) = 36.42, *p* < 0.001, *η*_*p*_^2^ = 0.12 and *F*(1282) = 5.50, *p* = 0.02, *η*_*p*_^2^ = 0.02, respectively), see also Fig. 3. Findings indicated reduced infrequent long response times in older compared to younger children.

Findings were similar when analyses were controlled for the influence of IQ. Covarying for inattention—but not hyperactivity/impulsivity—symptom severity attenuated the class main effect for *μ* (*p* = 0.17), suggesting severity of inattention was related to a stronger tendency to underproduce the 1-s interval. Covarying for other symptom domain measures did not influence the results.

Finally, as a check on the interpretation of our findings, analyses were repeated without unaffected siblings of ASD, ADHD, and ASD+ADHD-affected participants in the Normal class. This resulted in minor changes in outcome, which could not explain the absence of a difference between the ADHD-only class and the Normal class. Thus, the presence of unaffected siblings in the Normal class did not change the conclusions.

## Discussion

The present study examined whether reduced motor timing accuracy, increased timing variability, and infrequent long response times are specific for ADHD, or in part due to comorbidity with ASD. We compared motor timing difficulties across four homogeneous ASD–ADHD and unaffected latent classes derived from a clinic- and population-based sample. These homogeneous classes presented either no behavioral problems, purely ADHD behavior without any comorbidity, or both ASD and ADHD symptomatologies. In contrast to our hypotheses, the pure ADHD class did not deviate from the Normal class on any of the motor timing abilities (*μ*, *σ*, and *τ*). In fact, motor timing difficulties were found only in classes where both ADHD and ASD symptoms were present. The ADHD(+ASD) and ASD(+ADHD) classes showed a reduced motor timing *accuracy* (i.e., increased underproduction) compared to the Normal class. In addition, the younger children in the ADHD(+ASD) class had a reduced *variability* in motor timing when compared to the younger children in the Normal and ASD(+ADHD) classes, a pattern which was diminished across the older children.

The finding that the pure ADHD class did not deviate from the Normal class on the motor timing abilities may seem to contrast previous studies that used the same motor timing paradigm and found an increased tendency to underproduce time and an elevated motor timing variability in ADHD [9, 14, 28]. This contrast however is likely caused by the difference in groups across the studies; children who were DSM-defined as ‘ADHD’ may actually have suffered from comorbid ASD symptoms as well. Additionally, our pure ADHD class may have had milder problems than those typically included in case–control studies, suggesting that patients only with more severe behavioral symptoms show motor timing deficiencies. In our data, both the ADHD(+ASD) class (with highest ADHD symptoms), and the ASD(+ADHD) class (with highest ASD symptoms), differed from the pure ADHD and Normal classes. This shows that current motor timing results cannot merely be explained by high ADHD severity with ASD playing no role. That is, ADHD symptom severity in the pure ADHD and the ASD(+ADHD) classes were highly similar, while the former class showed no motor timing deficits. Controlling for inattention—but not hyperactivity/impulsivity—symptom severity attenuated the findings, suggesting that the severity of inattention symptoms seems most strongly related to the tendency to underproduce in this motor timing paradigm. Furthermore, the current finding parallels our previous study which indicated that homogeneous classes with children suffering from both types of symptoms were cognitively more impaired than children with pure ADHD symptoms, suggesting an overlapping cognitive background in ASD and ADHD [30]. The current findings are well in line with studies that found deficits in time processing in children with ASD regardless of ADHD comorbidity [17, 39]. It has been suggested that deficits in temporal processing interact with primary behavioral symptoms such as the poor development of social cognition in children with ASD [4]. Current underproduction of time across children and adolescents with both ADHD and ASD symptoms is also potentially related to primary real-life difficulties in planning and organizing tasks and task completion. For example, children and adolescents with ASD and ADHD may perceive the time set for a given (school) task as very long, and may overestimate the time that was needed to complete the task.

A recent meta-analysis on reaction time variability compared ADHD-affected children, adolescents (aged 13–18 years), and adults with clinical control groups [11]. Findings suggested that children but not adolescents with ADHD had a slightly elevated variability compared to the clinical control groups. In contrast, our findings suggest a reduced motor timing variability in the class of youngest children with ADHD(+ASD) symptoms. This reduced variability may reflect impulsivity or the tendency to rush a tedious task, one of the primary symptoms of ADHD. As discussed by Falter and Noreika [40], the interpretation of motor timing abnormalities in ASD and ADHD is obscured by the variety of tasks, modalities, exposure durations, and classifications used across studies. Our study adds important knowledge to this topic by reducing the clinical heterogeneity present in DSM-defined ASD and ADHD group comparisons. Our comparisons of motor timing abilities in empirically defined homogeneous ASD and ADHD classes suggest that ASD symptoms contribute to motor timing abnormalities. However, the role of ADHD in these combined classes is unclear, since a) no homogeneous class with only ASD symptoms emerged from the LCA, and b) the class with most severe ADHD symptoms presented with ASD symptoms as well [30]. In addition, the classes that presented with ASD symptoms also suffered from more symptoms on other behavioral domains such as oppositional behavior, anxiety, perfectionism, and emotional lability. Controlling for symptom severity in these domains did not however change the results. Although this profile of problems fits well with the symptom presentation of children with ASD and comorbid ADHD, it follows that no claim can currently be made regarding the *necessity* of ADHD symptoms for timing deficiencies to emerge when ASD symptoms are present.

Evaluation of the significance of timing differences and commonalities in pure ADHD and ASD with comorbid ADHD can be further elucidated by analyzing brain–behavior relationships. The extent to which substrates of timing related to pure ADHD are also related to ASD with comorbid ADHD, and vice versa, can increase our understanding of the role of time processing for the development of behavioral symptoms in ASD and ADHD. It has been suggested that time processing deficits in ASD are due to an abnormal cortical connectivity and synchrony as well as more diffuse and widespread neural abnormalities, with reduced volumes reported in the parietal lobe, limbic and cortical regions, and white matter tracts [41–43]. Functional magnetic resonance imaging (fMRI) data specifically focusing on the neural substrates of timing in children with ADHD indicated more confined deficits in the anterior cingulate gyrus, supplementary motor area, and their connections to fronto-striatal pathways [5]. Future fMRI studies across empirically defined homogeneous ASD and ADHD classes may be better apt to inform us on not only the neural mechanisms of timing, but also the possible shared and distinct substrates of timing in pure ADHD and comorbid ASD and ADHD. Given the ongoing debate on the association between timing deficits and specific ADHD symptoms [44, 45], future fMRI studies on the significance of relations between timing differences and commonalities in pure ADHD and ASD, and comorbid ADHD may benefit from actual behavior recordings (e.g., visual gaze) during actual timing tasks.

There are some limitations worthy of note. First, boys were overrepresented in the three affected classes, whereas they were underrepresented in the Normal class. This is because the symptoms of ASD and ADHD are more frequently seen in boys than in girls [46]. Note that this overrepresentation was present in all affected classes, and therefore did not affect comparisons between those classes. Second, the latent classes were based on questionnaires. In comparison with clinical interviews, questionnaires tend to overestimate the degree of comorbidity, as questionnaires do not allow for further probing or explanation of questions [47]. However, a possible overestimation of comorbidity cannot account for the differences in timing abilities in the distinct classes in the latent class analysis [30]. Third, the nature of our samples might have prevented us from detecting a homogeneous class with pure ASD behavior. ASD without ADHD symptoms might be underrepresented in clinic-based samples and rare in population samples. Therefore, very large samples are required to examine this issue further.
